# Two-stage ankle arthrodesis using the induced membrane technique for pyogenic arthritis: a case report

**DOI:** 10.1186/s13256-025-05110-8

**Published:** 2025-03-11

**Authors:** Ion Kimura, Youichi Yasui, Hirotaka Kawano, Wataru Miyamoto

**Affiliations:** Department of Orthopaedic Surgery, Teikyo University School of Medicine, 2-11-1, Kaga, Itabashi, Tokyo 173-8605 Japan

**Keywords:** Arthritis, Osteomyelitis, Ankle, Arthrodesis, Case report

## Abstract

**Background:**

Ankle arthrodesis is the most frequently performed salvage procedure for pyogenic arthritis. However, its failed fusion rate of approximately 15% has been considered problematic. Herein, we present a case of pyogenic ankle arthritis successfully treated via a two-stage surgical procedure on the basis of the induced membrane technique.

**Case presentation:**

A 43-year-old Japanese male patient with alcoholic liver disease was referred to our institution. He complained of persistent ankle pain and local heat following osteosynthesis for a closed pilon fracture. Radiological examinations revealed massive destruction of the ankle joint. Cultures of samples obtained from the joint isolated *Streptococcus* viridans. On the basis of these findings, he was diagnosed with pyogenic ankle arthritis with osteomyelitis of the distal tibia and talus. We performed the two-stage procedure per the induced membrane technique. In the first stage, the necrotic and infected tissue was debrided, and a polymethylmethacrylate spacer was inserted into the bone defect. Intravenous antibiotics were administered for 1 week thereafter. In the second stage, which was performed 5 weeks after the first stage, the induced membrane was identified around the polymethylmethacrylate spacer and cut to remove the latter. Ankle arthrodesis was performed with three double-thread screws. Finally, the autologous cancellous bone graft harvested from the ipsilateral iliac crest was used to fill the bone defect. During the postoperative period, antibiotics were administered intravenously for 2 weeks. Blood examinations normalized 3 weeks after the second stage. The immobilization splint was maintained for 6 weeks, after which partial weight bearing was started, and 6 months after surgery, the patient returned to full weight bearing and walked confidently without ankle pain. Radiological evaluations performed 2 years after the second stage revealed complete consolidation, and he reported no pain while walking.

**Conclusion:**

Ankle arthrodesis performed via the induced membrane technique not only successfully controlled infection, but also achieved complete bone union, enabling the preservation of ankle joint. This technique demonstrates its potential as a highly effective approach for treating pyogenic ankle arthritis.

## Background

Ankle arthrodesis for pyogenic ankle arthritis is the most frequently performed salvage procedure, and it aims to prevent more proximal amputation [1]. As the first-line surgical treatment prior to arthrodesis, debridement combined with local and/or systemic antibiotics is widely accepted [2]; then, internal and/or external fixation is performed. Despite several comparative studies [1, 3], the fixation methods for pyogenic ankle arthritis remain controversial, as a 10–15% failed fusion rate and a reinfection rate of ~ 20% have been considered problematic [1, 3]. These high failure and reinfection rates emphasize the need for alternative approaches.

The induced membrane technique (IMT) is a two-stage surgical procedure to reconstruct segmental bone defects due to osteomyelitis or open fractures of long bones [4]. First, necrotic and infected tissue is debrided, and a polymethylmethacrylate (PMMA) spacer is placed into the bone defect. Secondly, the removal of the PMMA spacer through the incised bioactive membrane induced by the first stage of surgery around the spacer, autologous cancellous bone grafting, and fixation are performed. Previous studies have reported an excellent prognosis of the IMT technique for the reconstruction of segmental bone defects in long bones [5]. Additionally, there have been several case reports on the application of IMT for arthrodesis [6–9]. However, to the best of our knowledge, no report has been published on the prognosis of the IMT technique for pyogenic ankle arthritis with osteomyelitis of the tibia and ankle thus far.

Herein, we present a case of pyogenic ankle arthritis accompanied by osteomyelitis of the tibia and talus, which underwent rapid destruction after osteosynthesis indicated for a pilon fracture. In this case, ankle arthrodesis was performed on the basis of the IMT technique, and the patient was able to return to social activity. This case highlights the novelty of applying the IMT for pyogenic ankle arthritis with osteomyelitis, demonstrating its potential as an alternative approach in challenging cases involving infection and significant bone loss.

## Case presentation (Table 1)

**Table 1:** Timeline of key events in the case based on referral to our institute as the baseline

Time (months since referral to our institute)	Event
−10	Initial injury (fall on stairs, diagnosed as pilon fracture)
−9.8	Osteosynthesis surgery
−7	Persistent pain noted; follow-up radiographs show gradual joint destruction
0	Referral to our institute
0.2	First-stage surgery (debridement, cement spacer insertion)
1.3	Second-stage surgery (spacer removal, bone grafting, internal fixation)
7.3	Patient begins full weight bearing
25.5	Complete consolidation (radiological confirmation)

### Initial presentation

Written informed consent was obtained from the patient for the publication of this case report and accompanying images.

**Fig. 1:**
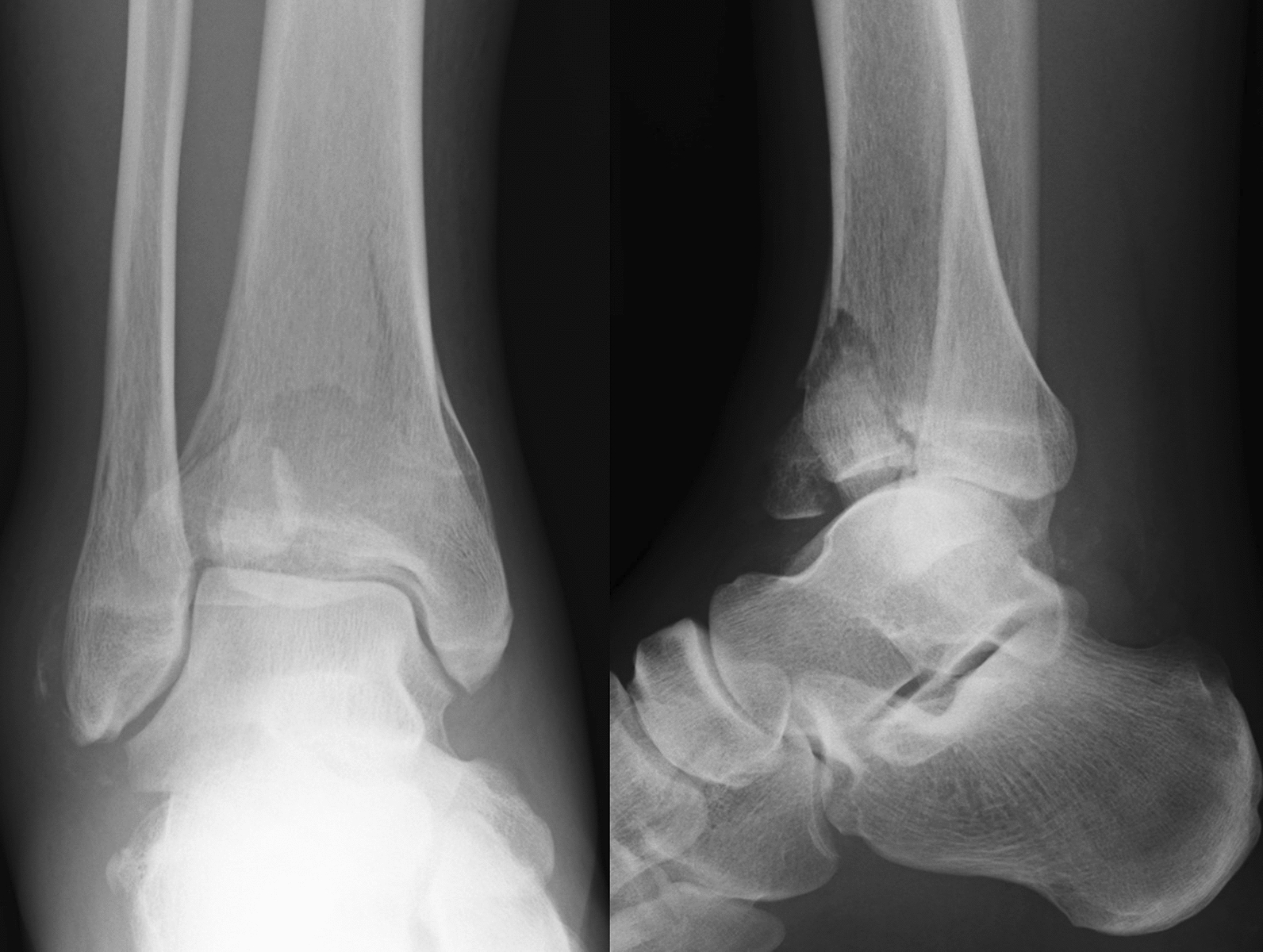
Plain radiographs of right ankle immediately after injury, showing a displaced pilon fracture: (right) anteroposterior view and (left) lateral view

**Fig. 2:**
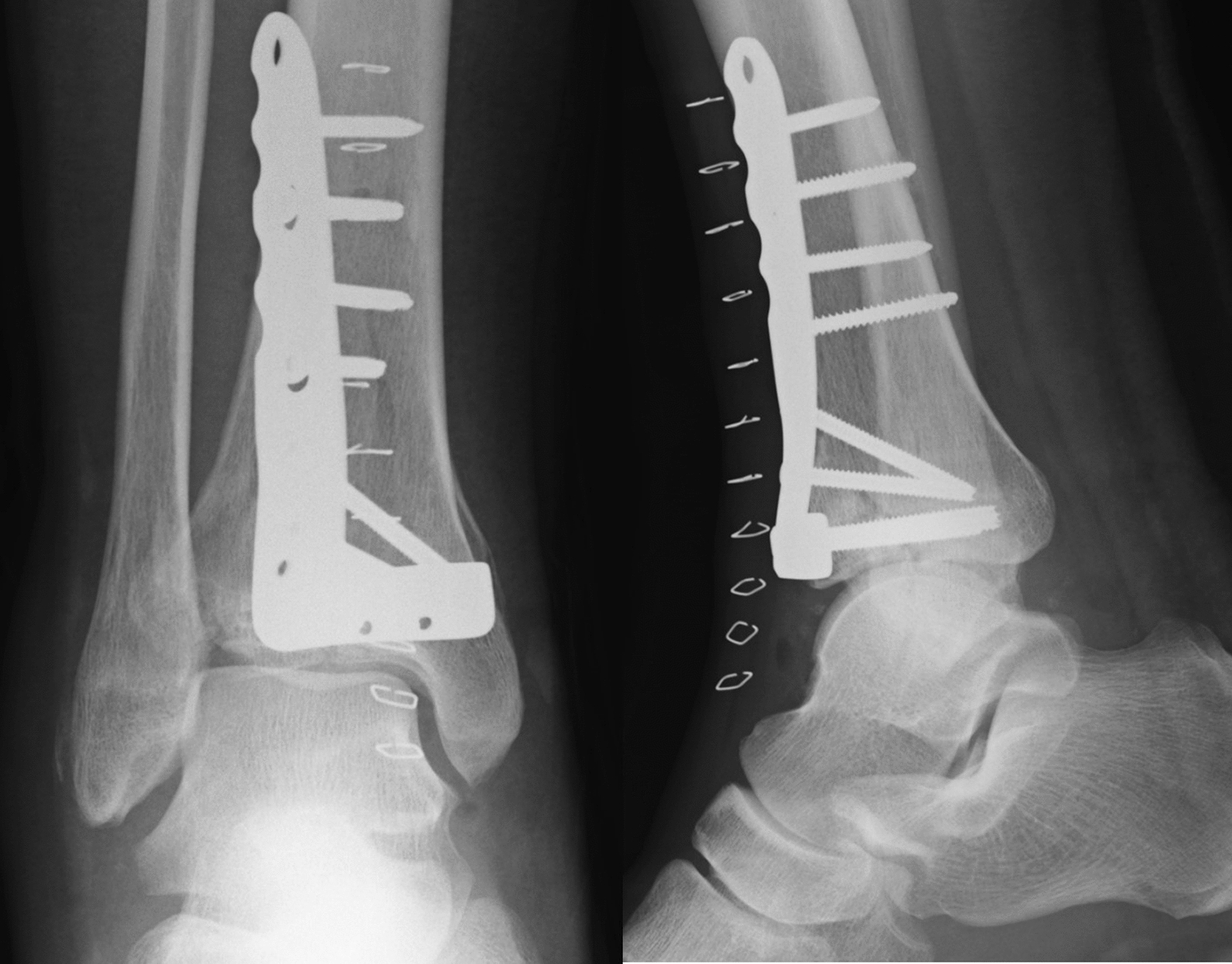
Plain radiographs of right ankle after the fixation; (right) anteroposterior view and (left) lateral view

**Fig. 3:**
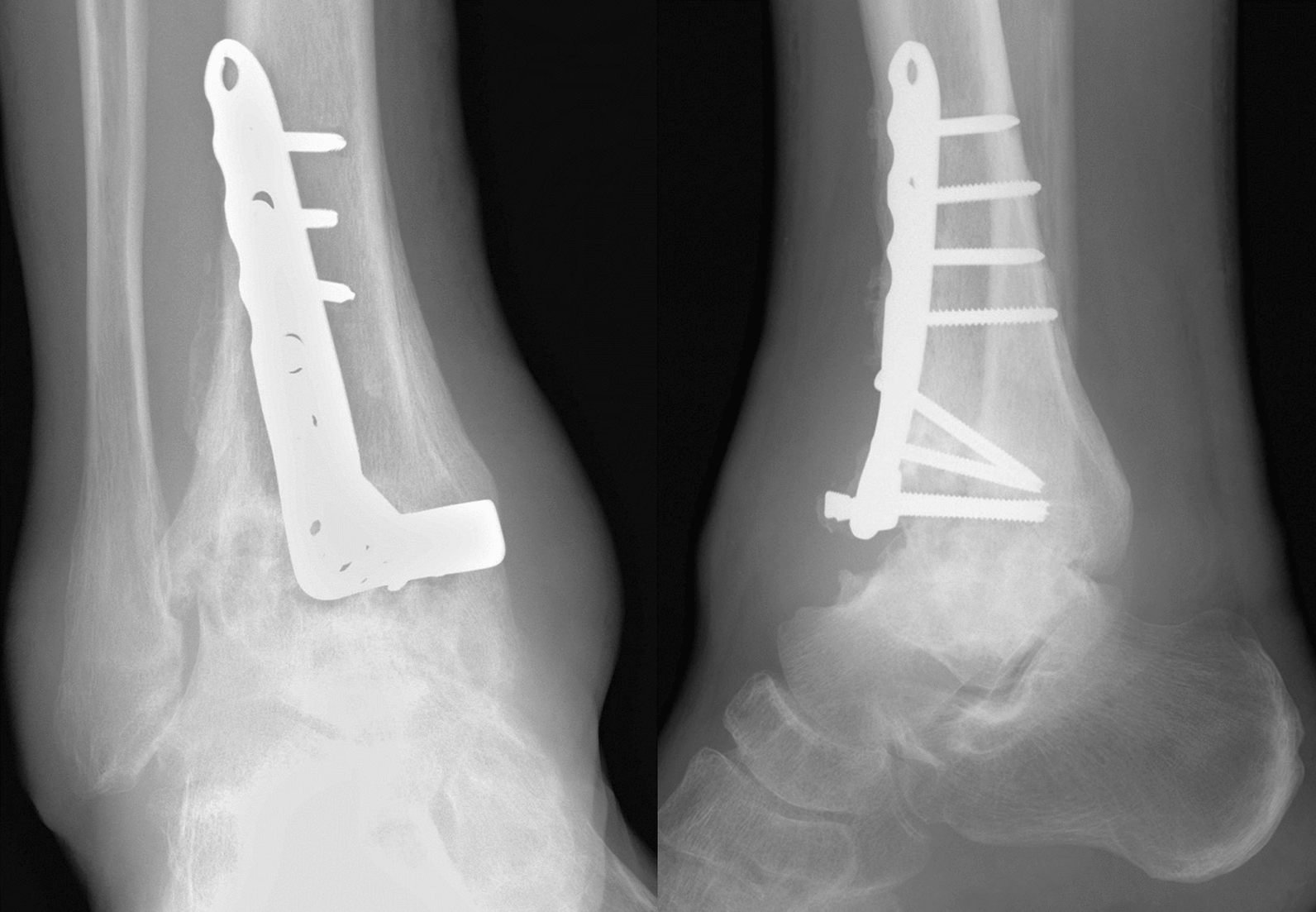
Plain radiographs of right ankle 10 months after osteosynthesis, demonstrating the rapid joint destruction subsequent to the procedure: (right) anteroposterior view and (left) lateral view

**Fig. 4:**
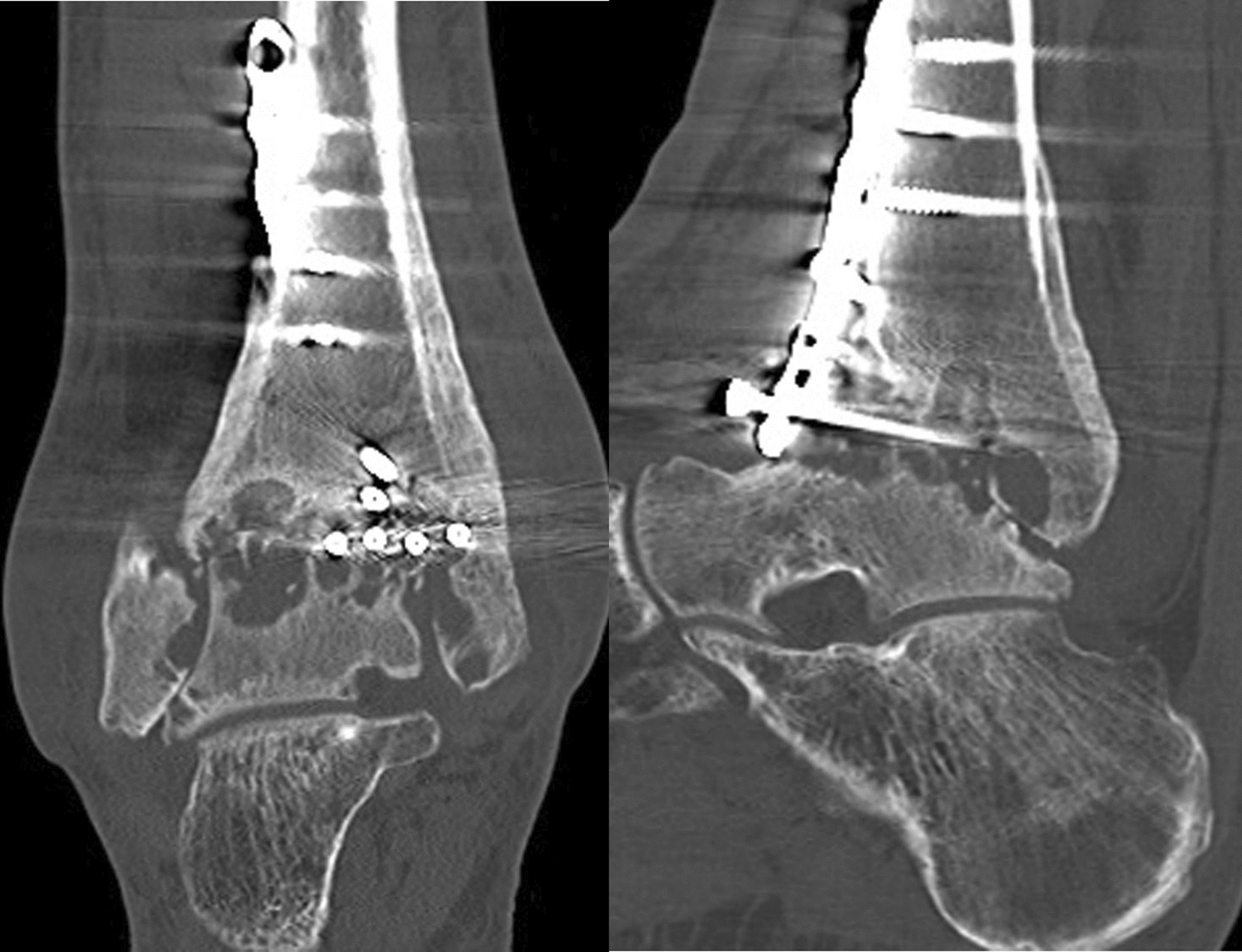
Plain computed tomography scan images of right ankle, presenting rapidly progressed joint destruction with extensive bone defects: (right) coronal view and (left) sagittal view

A 43-year-old Japanese male patient with a history of alcoholic liver disease who had persistent pain and swelling of his right ankle after osteosynthesis for a closed pilon fracture was referred to our institute. Before the onset of this condition, the patient was able to walk without any limitations. A total of 10 months earlier, he fell on the stairs and was immediately transported to a nearby orthopedic department where he was diagnosed with a right pilon fracture by plain radiographs (Fig. 1), and 5 days after the injury, he underwent osteosynthesis (Fig. 2). At 3 months after surgery, he was still unable to walk due to persistent severe ankle pain. Follow-up plain radiographs revealed the gradual destruction of his operated right ankle joint. Upon presentation at our institution, physical examination revealed local heat and tenderness over the ankle. The range of motion of the right ankle joint was restricted, with dorsal flexion of 5° and plantar flexion of 20°. Plain radiographs revealed the loss of joint space with screw backing out of the screw-in-plate (Fig. 3). Plain computed tomography (CT) scans revealed joint destruction with extensive bone defects (Fig. 4). Blood examinations revealed a slight elevation of the C-reacting protein level (0.83 mg/dl) and erythrocyte sedimentation rate (28 mm/h). On the basis of these findings, pyogenic arthritis of the ankle joint with osteomyelitis of the distal tibia and talus was suspected, and implant removal and synovectomy were performed. The culture of samples obtained from the resected synovium revealed the presence of *Streptococcus* viridans, and the clinical diagnosis was confirmed. To perform arthrodesis, two-stage surgery based on the IMT was planned.

### First-stage surgery

**Fig. 5:**
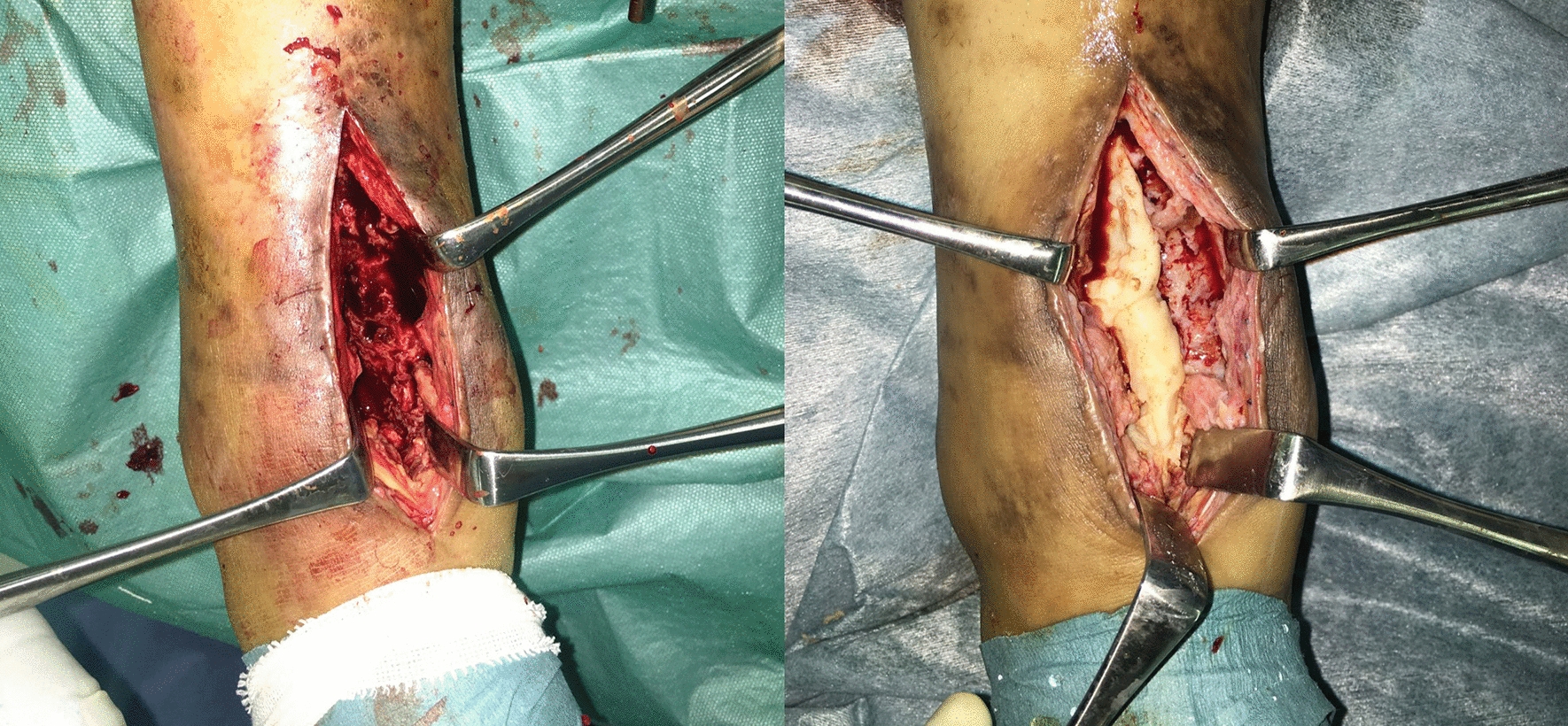
Intraoperative photographs in the first stage, (right) showing large bone defects with a longitudinal length of approximately 5 cm after aggressive debridement, and (left) showing the insertion of an antibiotic-impregnated PMMA spacer into the bone defect

The first stage of surgery, which consisted of aggressive debridement and the placement of a cement spacer into the bone defect, was conducted 11 months after the initial osteosynthesis. Under general anesthesia, necrotic and infected tissues were debrided to confirm the Paprika sign via an anterior approach. After aggressive debridement, a large bone defect with a longitudinal length of approximately 5 cm and the inferior part of the talus and posteromedial cortex of the distal tibia persisted. Next, 40 g of the PMMA spacer (Cemex® RX; Tecres Corp., Verona, Italy) were combined with 2.0 g of vancomycin powder, and then the antibiotic-impregnated PMMA spacer was filled up into the bone defect. (Fig. 5). After surgery, the ankle joint was immobilized in a neutral position, and daptomycin (490 mg/day) was administered intravenously for 1 week; 3 weeks after the first stage, blood tests showed normal levels of C-reactive protein, white blood cells, and erythrocyte sedimentation rates.

### Second-stage surgery

**Fig. 6:**
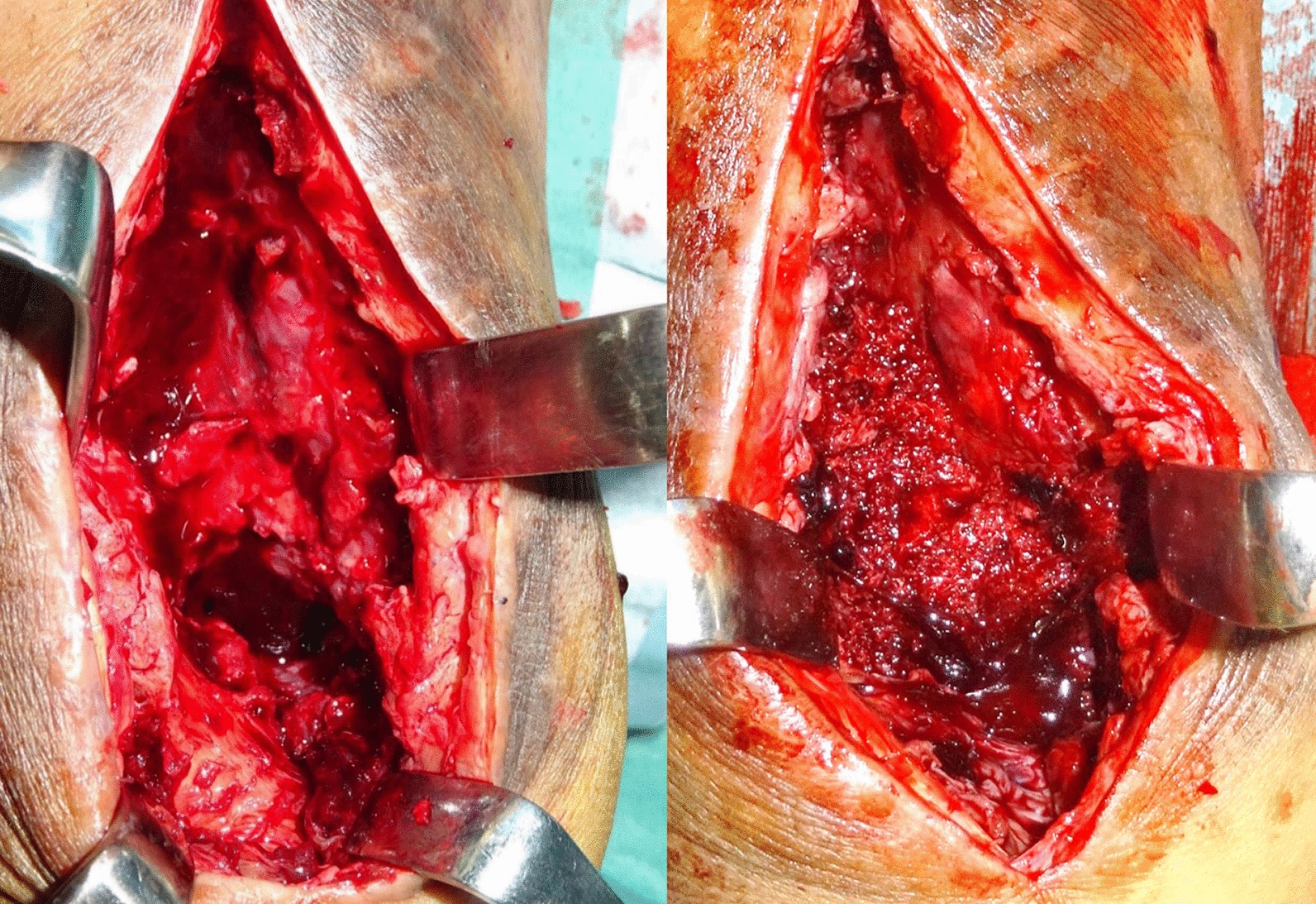
Intraoperative photographs in the second stage, (right) showing the bone defect after the removal of the PMMA spacer, and (left) showing filling of the bone defect with autologous cancellous bone harvested from the iliac crest after the internal fixation

Then, 5 weeks after the first stage, the second stage of the surgery, including the removal of the PMMA spacer, bone grafting, and internal fixation, was conducted. Under general anesthesia, the same skin incision was made as in the first surgery. The induced membrane over the PMMA spacer was split, and the spacer was removed. Internal fixation was then performed using three double-thread screws with a diameter of 7.0 mm with the ankle joint at approximately 5° of a dorsal flexed position. Subsequently, autologous cancellous bone harvested from the iliac crest was grafted to fill the bone defect (Fig. 6).

### Postoperative course

**Fig. 7:**
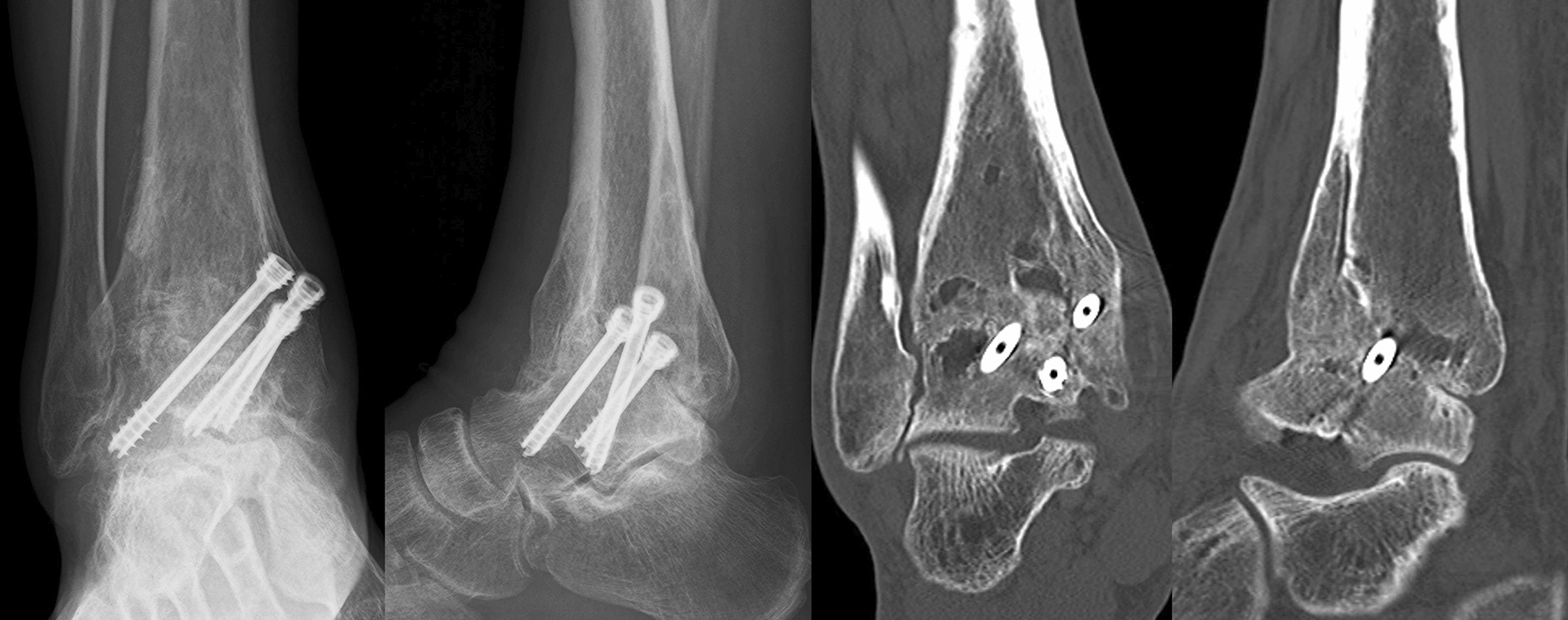
Radiological evaluation performed 2 years after the second stage, demonstrating complete consolidation after the ankle arthrodesis. Plain radiographs of the right ankle

After the second stage, linezolid (600 mg/day for 3 days), followed by daptomycin (490 mg/kg for 2 weeks), was administered intravenously. Routine blood examinations indicated normal C-reactive protein levels, white blood cell counts, and erythrocyte sedimentation rates 3 weeks after the second stage. The splint was kept for 6 weeks after the second stage, followed by the start of partial weight bearing. The patient began full weight bearing 6 months after the second stage, following our advice to proceed only when confident to walk without pain. Radiological evaluations performed 2 years after the second stage revealed complete consolidation, and he could walk without feeling pain (Fig. 7).

## Discussion and conclusion

Postoperative deep infection following osteosynthesis for a pilon fracture, which has a prevalence of 2–16%, is not rare [10]. Although it may be difficult to distinguish postoperative infection from hyperinflammation, delayed treatment may lead to pyogenic ankle arthritis accompanied by permanent joint destruction and osteomyelitis [2]. Unlike ankle arthrodesis for osteoarthritis, which results in bony fusion in more than 95% of cases [11], arthrodesis for pyogenic ankle arthritis using internal and/or external fixation results in nonunion in nearly 15% of cases [1, 3, 12]. Furthermore, it has been reported that about 20% of cases of reinfection occur after surgery [3]. The present case report describes a successful outcome of ankle arthrodesis for pyogenic ankle arthritis with osteomyelitis of the tibia and talus. This technique can represent a new surgical option for future patients and physicians who are managing similar cases.

The present case required aggressive debridement of the infected soft tissue and bones, resulting in significant bone defects around the talocrural joint. Traditionally, two surgical techniques have been applied for ankles with a significant bone defect. One of these is bone transport using Ilizarov techniques [13]. Its main advantage is the absence of limits concerning the size of the bone defect, while its disadvantages include prolonged external fixation, the resultant psychological burden on patients, frequent pin-tract infections, and the risk of a fracture of the regenerated bone [3, 4]. Another option is vascularized fibula graft transfer. A couple of reports demonstrated a good prognosis for ankle arthrodesis with significant bone defects [14, 15]; however, this technique requires expertise in microvascular surgery. Furthermore, there have been reported disadvantages, such as donor site morbidity, insufficient internal fixation, and restricted weight bearing for prolonged periods to prevent fracture during fibular hypertrophy [4]. 

The IMT was initially developed to treat segmental bone defects caused by osteomyelitis or open fractures in long bones. Several case reports have applied this technique for arthrodesis; however, all cases except one involved a small joint in the hand, foot, or elbow [6–8]. Regarding the ankle joint, Oh *et al*. first reported ankle arthrodesis using the IMT technique for an open fracture of the distal lower leg in 2019 [9]. Although their case involved no infection, arthrodesis based on the IMT technique was indicated because there was no possibility of reconstructing ankle function by osteosynthesis due to the segmental bone defect, and full weight bearing was achieved 18 months after the initial injury. To the best of our knowledge, this is the first case report on the application of the IMT for pyogenic ankle arthritis.

The main advantage of the IMT over bone transport or vascularized fibula graft transfer is that it is a less technically demanding procedure for ankle arthrodesis. While this study does not include a detailed cost analysis, the IMT’s technical simplicity and reduced need for specialized equipment may make it a more accessible option for general orthopedic surgeons worldwide. The IMT includes debridement of infected tissues, internal fixation, and autologous bone grafting, and all such procedures can easily be performed by general orthopedic surgeons without special skills. Regarding the fixation technique, screw fixation, which is the standard procedure for ankle arthrodesis, was applied. In the present case, despite the significant bone defect following aggressive debridement, screw fixation was conducted because the inferior part of the talus and posteromedial cortex of the distal tibia were preserved after debridement. If screw fixation is not feasible, retrograde nailing, which injures the subtalar joint, might be considered.

We reported the effectiveness of ankle arthrodesis on the basis of the IMT technique for pyogenic ankle arthritis; however, further studies evaluating more cases in which this procedure was used are necessary to establish this procedure as the gold standard for pyogenic ankle arthritis. Although this case demonstrated no evidence of reinfection, it should be noted that reinfection remains a critical concern in procedures addressing pyogenic arthritis, especially in cases involving large bone defects. Moreover, the long-term outcomes of IMT for pyogenic ankle arthritis remain unknown, and further research is required to evaluate its durability and efficacy in broader clinical settings. Potential challenges, including the optimal timing of stages and anatomical variations, warrant consideration in future research.

## Data Availability

Not applicable.

## References

[1] Suda AJ, Richter A, Abou-Nouar G, Jazzazi M, Tinelli M, Bischel OE. Arthrodesis for septic arthritis of the ankle: risk factors and complications. Arch Orthop Trauma Surg. 2016;136(10):1343–8. 27447881 10.1007/s00402-016-2520-y

[2] Movassaghi K, Wakefield C, Bohl DD, *et al*. Septic arthritis of the native ankle. JBJS Rev. 2019;7(3): e6. 30889008 10.2106/JBJS.RVW.18.00080

[3] Rüschenschmidt M, Glombitza M, Dahmen J, Hax P-M, Lefering R, Steinhausen E. External versus internal fixation for arthrodesis of chronic ankle joint infections—A comparative retrospective study. Foot Ankle Surg. 2020;26(4):398–404. 31129101 10.1016/j.fas.2019.05.001

[4] Mauffrey C, Barlow BT, Smith W. Management of segmental bone defects. J Am Acad Orthop Surg. 2015;23(3):143–53. 25716002 10.5435/JAAOS-D-14-00018

[5] Giotikas D, Tarazi N, Spalding L, Nabergoj M, Krkovic M. Results of the induced membrane technique in the management of traumatic bone loss in the lower limb: a cohort study. J Orthop Trauma. 2019;33(3):131–6. 30562247 10.1097/BOT.0000000000001384

[6] Flamans B, Pauchot J, Petite H, *et al*. Use of the induced membrane technique for the treatment of bone defects in the hand or wrist, in emergency. Chir Main. 2010;29(5):307–14. 20728395 10.1016/j.main.2010.06.008

[7] Rincón-Cardozo DF, Camacho-Casas JA, Reyes-Núñez VA. Dislocation and necrosis of the first, second and third wedges: management with the Masquelet technique—A case report. Acta Ortop Mex. 2013;27(1):55–9. 24701753

[8] Alassaf N, Alhoukail A, Alsahli A, Althubaiti G. Salvage of mangled upper extremity using the Masquelet technique in a child: a case report. SAGE Open Med Case Rep. 2017;16(5):2050313X17741011. 10.1177/2050313X17741011 PMC5697582 29201370

[9] Oh Y, Yoshii T, Okawa A. Ankle arthrodesis using a modified Masquelet induced membrane technique for open ankle fracture with a substantial osteochondral defect: a case report of novel surgical technique. Injury. 2019;50(11):2128–35. 31530381 10.1016/j.injury.2019.09.020

[10] Molina CS, Stinner DJ, Fras AR, Evans JM. Risk factors of deep infection in operatively treated pilon fractures (AO/OTA: 43). J Orthop. 2015;12(Suppl 1):S7–13. 26719630 10.1016/j.jor.2015.01.026 PMC4674535

[11] Kennedy JG, Hodgkins CW, Brodsky A, Bohne WH. Outcomes after standardized screw fixation technique of ankle arthrodesis. Clin Orthop Relat Res. 2006;447:112–8. 16741477 10.1097/01.blo.0000203480.04174.0e

[12] Klouche S, El-Masri F, Graff W, Mamoudy P. Arthrodesis with internal fixation of the infected ankle. J Foot Ankle Surg. 2011;50(1):25–30. 21172639 10.1053/j.jfas.2010.10.011

[13] Atef A, El-Rosasy M, El-Tantawy A. Salvage arthrodesis for infected ankle fractures with segmental bone-loss using Ilizarov concepts: a prospective study. Int Orthop. 2021;45(1):233–40. 33196902 10.1007/s00264-020-04874-6

[14] Bumbasirevic M, Stevanovic M, Bumbasirevic V, Lesic A, Atkinson HD. Free vascularised fibular grafts in orthopaedics. Int Orthopb. 2014;38(6):1277–82. 10.1007/s00264-014-2281-6 PMC4037521 24562850

[15] Piccolo PP, Ben-Amotz O, Ashley B, Wapner KL, Levin LS. Ankle arthrodesis with free vascularized fibula autograft using saphenous vein grafts: a case series. Plast Reconstr Surg. 2018;142(3):806–9. 30148787 10.1097/PRS.0000000000004671

